# Reflections of older people about their experience of fall prevention exercise in the community- a qualitative study exploring evidence-based practice

**DOI:** 10.1186/s12889-020-09630-4

**Published:** 2020-11-09

**Authors:** Hilde Worum, Daniela Lillekroken, Kirsti Skavberg Roaldsen, Birgitte Ahlsen, Astrid Bergland

**Affiliations:** 1 Department of Physiotherapy, Faculty of Health Sciences, Oslo Metropolitan University, Oslo, Norway; 2 Department of Nursing and Health Promotion, Faculty of Health Sciences, Oslo Metropolitan University, Oslo, Norway; 3 Department of Neurobiology, Health Sciences and Society, Karolinska Institute, Stockholm, Sweden; 4 Department of Research, Sunnaas Rehabilitation Hospital, Oslo, Norway

**Keywords:** Collaboration, Engagement, Fall prevention, Impact, Knowledge translation, Patient and public involvement

## Abstract

**Background:**

Evidence-based practice (EBP) ensures that clinicians use effective interventions to achieve desired outcomes, thereby contributing to the best quality of care. The perspective of the participants is fundamental in EBP, as they have their own individual and meaningful rationale for participating in fall prevention. This study aims to explore community-dwelling older people reflections about their reflections about EBP in physiotherapy based on their experiences of a fall prevention exercise program.

**Methods:**

We conducted semi-structured interviews with 16 community-dwelling older people (men = 7; women = 9). Data were analyzed using thematic analysis.

**Results:**

The analysis revealed three themes: 1) the tension between knowing and doing, 2) the power of the therapist-participant relationship and the process of putting knowledge into action, and 3) research is interwoven with successful therapy and is an integral component of it. EBP was considered as a collective negotiation and learning process of creating knowledge for clinical practice. The negotiation between different types of knowledge must be performed in a transparent dialogue and through interactive collaboration between the persons involved. The participants appreciated that the research findings indicate that practice gives results.

**Conclusions:**

EBP was understood and utilized as a seal of approval and a “guarantee of high quality” treatment, and its effects varied based on older people’s preferences, needs, and skills. The therapist’s relational competence appeared to be crucial for the negotiation of various sources of knowledge relative to the older people’s preferences.

## Background

**Fig. 1:**
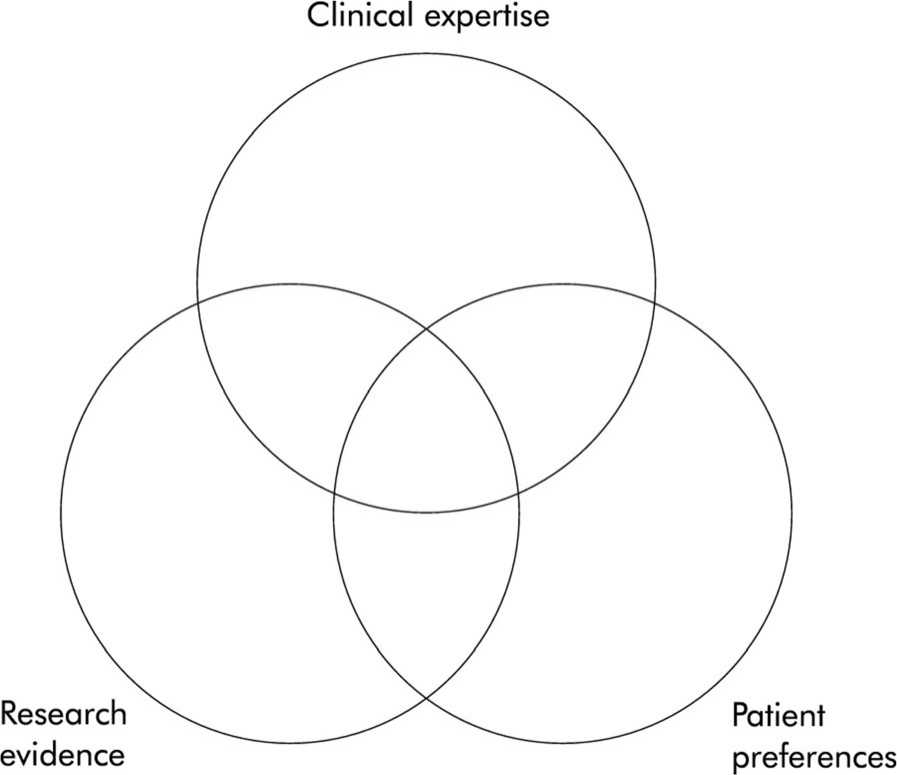
Evidence-based practice. Haynes R.B., Devereaux P.J., Guyatt G.H. 2002. Clinical expertise in the era of evidence-based medicine and patient choice. BMJ Evidence-Based Medicine [46]. Permission were obtained from Brian Haynes 14.01.2020

Due to the increasing proportion of older people in society [1], efficient and effective evidence-based practice (EBP) strategies for managing fall prevention in primary health care are of great importance. Offering customized health services as people become older is a common challenge across Europe [2]. EBP aims to integrate the best available evidence, clinical expertise, and patient values into clinical practice to ensure positive patient outcomes [3], as illustrated in Fig. 1. Applying EBP in primary health care settings ensures that clinicians use effective interventions to achieve desired outcomes, thereby contributing to the best quality of care [4]. However, there are several barriers for EBP and the components separately (evidence-based knowledge, clinical expertise, and patient values within a given context) that must be overcome in order to succeed with increased quality of care. Despite the clear benefits of EBP in fall prevention [5, 6], translating evidence into clinical practice has been challenging [7]. Although falls are preventable [5, 6], the incidence of falls has not been reduced [8], and uptake rates of evidence-based interventions in community settings are low [9]. Iles and Davidson [10] found that physiotherapists’ main barriers to EBP were lack of access to easily understandable summaries of evidence, limited journal access and a lack of skills in searching and evaluating research evidence. Features of evidence-based knowledge have been reported to represent difficulties because of the attributes related to the research articles, for example, ease-of-use, relevancy and applicability in practice, understood as the readability of research articles, formulations and style of the recommendations, including their complexity [11]. EBP is largely influenced by contextual conditions that also include conditions outside the clinical setting. Factors at the macro level have appeared to be a tremendous barrier to fall prevention. Lack of financing and reimbursement of Medicare and private medical insurance has hindered providers’ ability to offer sufficient fall risk assessments and refer patients to an adequate intervention [12]. Moreno-Peral et al. [13] claimed that public and private health organizations do not promote primary prevention and health promotion activities for fall risk management because they are considered to be unprofitable. Furthermore, frequently reported barriers for the organizational context level has been too few staffing resources, and lack of time. Results from a recent study demonstrate that institutional barriers including workload, demands of routine clinical practice, and time limitations are also barriers to fall prevention management [12]. The cause of the barriers is practically linked to high patient loads and time constraints [14], as well as overwhelming amount of guidelines [15]. Health care professionals’ view fall prevention as important but are lacking the knowledge to integrate the guidelines into practice [16]. Providers do not feel sufficiently informed to educate their participants on fall prevention or conduct a fall risk assessment [17]. The last and perhaps the most crucial component is the main character, the older person. Older people’s knowledge, behavior, beliefs, skills, motivation, and resources can influence their attitudes and contribute to making favorable or not favorable health decisions [13]. Many older people reject the risk of falling due to a perceived association with dependency and incompetence. The consequences are that older people fail to participate in fall prevention interventions [18]. However, there are older people who accept the risk of falling and view falls as something that negatively affects confidence, independence, and quality of life [19]. Laing et al. [20] noted that motivation to participate in fall prevention interventions, occurred after older adults had experienced a fall, which increased their perceived risk. Evidence-based interventions have shown to reduce falls for those who take part. However, they can only prove effective at a population level if participation rates are high [21]. Furthermore, the effectiveness of evidence-based fall prevention is only as strong as the level of adherence to those recommendations [22].

The Canadian Institutes of Health Research define knowledge translation as *“a dynamic and iterative process that includes synthesis, dissemination, exchange, and ethically sound application of knowledge to improve the health of Canadians, provide more effective health services and products, and strengthen the health care system”* [23]. This process takes place within a complex system of interactions between researchers and knowledge users, which may vary in intensity, complexity, and level of engagement, depending on the nature of the research and the findings, as well as the needs of the particular knowledge user [24]. The common feature of all knowledge translation processes is that they are concerned with conditions that inhibit and promote the introduction of new evidence-based knowledge in clinical practice [24, 25]. A range of factors related to older people who have experienced falling, families, health care professionals, and health care systems affect the knowledge translation of fall-prevention interventions [8, 19, 26, 27]; therefore, it is important to consult with older people and professionals in order to ascertain what changes they are prepared to make to reduce the risk of falling. If this consultation does not happen, then fall-prevention interventions may be less effective, as the most feasible and relevant interventions may not be targeted, and the maximum participation rates may not be achieved [19].

The perspective of older people is fundamental in EBP [25, 28, 29], as they have their own individual and meaningful rationale for participating in interventions [30]. Considering the views of older people is important for identifying their needs, which in turn helps providers to develop appropriate and responsive services [31]. McMahon et al. [31] suggested that, by exploring individual challenges and perspectives about fall risk and participation in fall-prevention programs, nurses could build stronger partnerships with older people and develop person-centered approaches to care. Person-centred practice is defined as a holistic approach (seeing the person behind the diagnosis) characterized by respect and empathy, with an emphasis on the persons’ meaningful life and capabilities [32, 33]. Recognizing the views of older people receiving evidence-based fall-prevention intervention might be the first step towards participating in and adhering to EBP. According to Finnegan et al. [30], it is important that health care professionals get to know older people’s rationale for participating in EBP training in order to better support these individuals as they adhere to structured fall-prevention exercises. Public and patient involvement (PPI) in clinical decision-making allows for active contribution to research design, acceptability, relevance, conduct, governance, and knowledge dissemination [34].

A recent qualitative study examined older people’s and physiotherapists’ views on the factors that influence the implementation of the evidence-based Otago Exercise Programme for fall prevention, and their views on evidence-based knowledge [25]. The older people perceived the Otago Exercise Programme as meaningful, effective, and safe. The findings underscored the need for clinicians and researchers to understand the importance of user-friendly language, and to contextualize research in terms of clinical expertise and the older people’s values. This study focused primarily on the physiotherapist’s point of view on the implementation of evidence-based knowledge. Studies on older peoples’ perspectives have highlighted factors that influence uptake and attitudes to fall prevention exercise programs among older people [31, 35, 36]. However, while other researchers have mainly focused on the older people’s perspectives on fall prevention, limited to the actual training or treatment, we are interested in older peoples’ reflections and perspectives on the three components of EBP - evidence-based knowledge, clinical expertise, and patient values- in fall-prevention. Currently, there is limited knowledge about older people’s views on EBP in fall prevention, including evidence-based knowledge, clinical expertise and patient values. Though older people are the end-users of EBP, their views and perspectives have received limited attention in implementation science [37], which is often used synonymously with knowledge translation [24]. Minogue et al. [38] suggested that involving “users” in research would reduce wasted research and enhance the quality of fall-prevention initiatives. According to Tomlinson et al. [39], PPI in research may improve the quality of research projects and strengthen the relevance and impact of research. In addition to these benefits, participants and members of the public reported that they were provided with a mechanism to share their experiences while directly influencing change, empowering them to contribute to society and gain new skills [39].

The aim of this study was to explore community-dwelling older peoples’ reflections and experiences about EBP in physiotherapy regarding fall prevention exercise, so as to contribute to close the know-do gap in fall prevention. Understanding the views and expectations of older people is fundamental as well as facilitating person-centered care, because respect for individual diversity is a basic pillar in the approach of EBP [40]. We wanted to explore, from the older peoples’ perspective, the relations between the three dimensions of the EBP model: evidence-based knowledge, clinical expertise, and patient values. We did this in the context of Norwegian primary health care. The following research question was formulated: what are older peoples’ reflections and perspectives on the three components of EBP—evidence-based knowledge, clinical expertise, and patient values—in fall-prevention intervention? Older peoples’ opinions will help us to understand their aspirations, values, and decisions, thereby providing health and social care professionals with a common understanding of the principles of appropriate individual health care [41]. It is expected that the findings of this study will contribute to inform policy makers, educators, clinicians, future researchers, and older adults and thus improve implementation of EBP in fall prevention. The current paper is the first of a two-part report featuring discussions with both older peoples’ and physiotherapists about their reflections on and experiences of EBP in fall prevention.

## Methods

To answer the research question, we employed a qualitative approach using individual semi-structured interviews, as described by Creswell and Poth [42]. Given that one must be open to lived experiences in order to see things as they are, we sought to gain a deeper insight into the research phenomena by adopting a phenomenological approach [42]. A phenomenological perspective explores how human beings make sense of experiences and transform those experiences into consciousness, both individually and as a shared meaning [43]. A phenomenological perspective incorporates the perceptions and feelings of people associated with what they experience, not merely the observations of the experience itself [42]. The goal is to summarize individual experiences and provide descriptions that include “what” people experience and “how” they experience it [44].

### Recruitment and participants

We recruited a purposive sample. A purposive sample selects participants that can best address the research problem and inform an understanding of the phenomenon under study [42]—that is, participants with experiences relating to the phenomena being studied. Inclusions criteria were: 1) individuals older than 65 years of age, 2) who had experienced a fall, and 3) who had participated in a fall-prevention intervention in their respective regions provided by a physiotherapist. Fall-prevention intervention was defined as a structured, targeted exercise program—in other words, a treatment measure with a set start/end time that is offered to older peoples who have experienced a fall. The study included older peoples who participated in individual and group interventions. The older people in this study were recruited through primary health care services from four different regions in Oslo, Norway. The participants’ former physiotherapists in the respective regions were responsible for the recruitment and were informed about the purpose of the study, as well as procedures for data collection. The physiotherapists ensured the ethical integrity of the study, informing each participant of their ability to consent and providing them with the name of the first author. After the physiotherapist assessed the older people who were considered as potential participants, the first author contacted them by telephone and invited them to participate in the study. The first author provided them with both written and verbal information about the study. Recruitment was considered complete when theoretical saturation was achieved, i.e., when the interviews provided no new information. All the participants delivered detailed descriptions, and data were deemed to be rich enough to elaborate a general structure.

In total, 16 older people (men = 7; women = 9) were included, and data saturation was achieved after 14 interviews. The participants had participated in either an individual or a group intervention. The participants ranged in age from 68 to 93 years (mean age 80 years), with a mean score of 8.75 on the Short Physical Performance Battery (SPPB) [45]. SPPB is a common, well-established instrument for measuring physical performance. It involves a timed 4-m walk, timed and repeated sit-to-stand test, and 10-s balance tests (side-by-side, semi-tandem, and full-tandem) [45]. The timed results of each subtest are rescaled according to predefined cut points to obtain a total score, which ranges from 0 (worst performance) to 12 (best performance) [45].

### Data collection

Between May 2019 and September 2019, the first author conducted 16 semi-structured interviews. All interviews were conducted according to an interview guide based on the theory and rationale behind the concept of EBP [3]. We had a particular focus on user involvement, or how the older people experience making decisions about their treatment. We assumed that the information would enhance our understanding of older peoples’ attitudes about EBP.

### Interview guide

The interview guide developed for this study was created in collaboration by all authors (see Additional file 1). The interviews were initiated using open-ended questions inviting the older people to talk about their general experiences with the intervention, followed by more specific questions related to the dimensions of the EBP model—evidence-based knowledge, clinical expertise, and patient values—and how they interact with each other. Examples of questions from the interview guide can be seen in Table 1. The participants were provided with an illustration of the EBP model as a visual tool to better understand the questions during the interview (Fig. 1). The interviews were conducted in the participants’ homes and lasted for 45–60 min, depending on the participants’ involvement. All interviews were audiotaped. The quotes were translated from Norwegian into English by the first author.

**Table 1:** Examples of questions in the interview guide

•Can you please describe your experience of participating in the exercise intervention from start to finish? •Can you please describe your confidence in research on fall prevention? •Can you please describe qualities of a good therapy? •Can you please describe what motivates you to participate in fall-prevention intervention? •Is there anything else you would like to share?

### Data analysis

The research group consisted of females with a background in nursing and physiotherapy. Except for the first author, who is a PhD student, the researchers are associate professors or professors and participated in the analysis. The interviews were transcribed verbatim by an external research assistant. The qualitative data analysis framework used was based on Braun and Clarke’s thematic analysis [47]. This is an analytical approach used to find patterns across data sets. Within the thematic analysis, an inductive approach was adopted in which themes and codes emerged from the data itself. The inductive approach enables researchers to identify key themes in the area of interest by reducing the material to a set of themes or categories. Themes were identified at the latent level and examined the underlying assumptions or ideas behind participants’ descriptions of their participation in an EBP fall-prevention intervention. The analysis was carried out in the following six steps: 1) familiarization with the data, 2) generating initial codes, 3) searching for themes, 4) reviewing themes, 5) defining and naming themes, and 6) producing the report. Examples of the coding strategy are presented in Table 2. To maintain rigor throughout the analysis, features of trustworthiness were established according to Lincoln and Guba’s criteria of soundness [48]. The criterion of credibility was met through open-ended questioning and prolonged engagement with the data, and by providing a detailed description of the methods. The criterion of transferability was fulfilled by presenting detailed and in-depth descriptive data from the participants’ quotes. To meet the criterion of dependability, each transcription was independently read, checked, and coded by the first and the last authors; final interpretations were reached via agreement among all five authors. The criterion of confirmability was fulfilled by providing rich quotes from the participants depicting each emerging theme. Furthermore, the Consolidated criteria for reporting qualitative research (COREQ): a 32-item checklist for interviews and focus groups (Additional file 2) [49] and Guidance for Reporting Involvement of Patients and the Public (Additional file 3) [50] were considered for reporting the current study.

**Table 2:** A summary of the emergent sub-themes, themes, and core theme

Examples of participants’ responses	Code	Sub-theme	Theme
*I’m not sitting in this chair and nodding off so much, I have a little more energy and I think I have a little more appetite. For some reason, I have lost some appetite over the last few years, but it has come back a bit now. I can feel I’m hungry now, for a while I didn’t notice it. So, there was a time when I stopped exercising and sat nodding off, had no appetite...it’s gotten a little better now* (Participant ID 1)	•Inactivity/sedentary behavior •Energy •Positive change/experience	The older person’s desire to cope with everyday life	The tension between knowing and doing
*It wasn’t out of great desire or eagerness from me, but I am so sensible and there is so much health in it ... I have told the physiotherapist that it is not out of desire and interest that I am here, but I realize I need to do something* (Participant ID 4)	•Unmotivated •Positive change/experience •Reality check •Motivation to exercise	Acquiring knowledge; knowledge as the basis for evidence-based practice	
*Empathy is important too; without it something may go wrong. You have to get into things - me and the neighbor may want to experience things completely differently, and we would have different needs even if we were dealing with the same illness … and it is perhaps what a therapist with experience will find out more easily than a recently graduated one?* (Participant ID 2)	Importance of knowledge •Diversity/heterogeneity •Different needs •Importance of therapist’s experience/the therapist as a human expert •Getting into things	Evidence balanced by understanding, empathy, and sympathy	The power of the therapist-participant relationship and the process of putting knowledge into action
*He was so safe and secure when he gave us assignments and it is contagious. He always said: this is not dangerous; you can do this ... it seemed very safe ... I trusted him and what he taught me* (Participant ID 14)	•The therapist’s experience •Safety •Knowledge •Relation •Communication	The importance of mutual understanding	
*I have a son who is mentally ill … we tend to walk almost every day … his biggest problem is that he is isolating himself, and what I can contribute is to get him out for a walk ... what really motivates me is that I want to stay in the best possible shape since I have a son that I feel is dependent on me* (Participant ID 2)	•Everyday activities/providing care •Transferability of exercise •The importance of contribution •Effectiveness of intervention	The older persons’ desire to cope with everyday life	Research is interwoven with successful therapy and is an integral component of it
*If someone had said do that and that, and I didn’t realize the point, I wouldn’t have bothered to do it ... waving arms and legs with no intention is just nonsense* (Participant ID 2)	•Explanation of the exercises •Power •Motivation •Making sense •Communication	In clinical practice, research makes sense	

### Ethical considerations

The study was approved by the Norwegian Ethics Committee for medical and health research ethics (2018/2227/REC south-east C). The physiotherapists from the different regions in Oslo, who were responsible for initial contact with the older people, were informed about the study in writing and verbally. Only older people with the ability to consent were recruited. The first author contacted the older people by phone and scheduled the day and the time for an interview. Time and place of the interview were organized according to the participants’ convenience. Information about the study, including the right to withdraw from the study at any time as long as the data were not analyzed, was provided both during recruitment and at the time the interview took place. It was emphasized that participation was voluntary and that the participants were guaranteed confidentiality and anonymity in the process and in subsequent publications. All the older people included in the study gave their written informed consent to participate in the study.

## Results

Totally 16 older people were interviewed. Table 3 describes the characteristics of the participants.

**Table 3:** Characteristics of the participants

Participant ID	Gender	Age	Education level	Living alone (Yes/ No)	Number of medications	SPPB score	Walking aid (Yes/ No)	Diagnosis (Number)
1	M	68	High	No	3	8	Yes	3
2	F	75	High	Yes	6	10	No	2
3	M	81	Middle	No	1	11	Yes	None
4	M	83	High	Yes	3	12	No	None
5	F	78	Middle	No	3	9	Yes	1
6	F	88	Middle	Yes	8	6	Yes	3
7	F	78	Middle	No	4	11	Yes	4
8	F	74	Middle	Yes	4	11	Yes	2
9	M	77	High	No	0	9	No	None
10	F	76	Middle	Yes	3	9	Yes	2
11	M	76	Middle	No	16	1	Yes	4
12	M	91	High	No	5	9	No	3
13	M	89	Middle	Yes	6	10	Yes	2
14	F	90	Middle	Yes	5	5	Yes	2
15	F	93	High	Yes	4	8	Yes	3
16	F	69	High	Yes	0	11	No	None

Note: *F* female, *M* male, Education level: low (7 or less years), middle (8–12 years), or high (13 or more years), SPPB score: summed score for the Short Physical Performance Battery

The analysis revealed the following three themes: 1) the tension between knowing and doing, 2) the power of the therapist-participant relationship and the process of putting knowledge into action, and 3) research is interwoven with successful therapy and is an integral component of it.

KT is a dynamic and contextual process involving several forms of knowledge, wherein knowledge is translated from research into practice, resulting in best practice for a given context. The negotiation between different types of knowledge must be performed transparently, and in a way that allows participants to be conscious of complexity before making decisions about their own treatment based on research discussions, knowledge exchange, and experience. Emphasis should be placed on the interactive collaboration between the persons involved, and on the social and material dimensions involved in the process of sense-making. EBP should be recognized and confirmed by others, for example, through their acceptance and adaption of a useful solution or a practical way of reasoning. This validation requires close dialogue between the clinical field and its participants, physiotherapists and their participants, which poses a big challenge for the future.

### The tension between knowing and doing

The participants expressed different views on EBP regarding their preferences and values as well as the three components separated. For some, knowledge about the treatment and desire to be able to live as long as possible, or to age without functional limitations, were highlighted as motivational factors for exercise. For most of them, having clear and defined goals was important for maintaining motivation, or for “making sense” of the intervention and the value of engaging in it, relative to the challenges of everyday life. Explanations of the rationale behind the exercises were also described as crucial for older people’s engagement in the intervention. Along with determination, professional knowledge about fall-prevention and exercise becomes a trump card against ambivalence and resistance to exercise. Older peoples’ preferences, knowledge, motivation, abilities, and skills influence them to varying degrees to make important health-related decisions and are therefore crucial for their participation in the intervention.

#### The older person’s desire to cope with everyday life

According to the participants, to succeed with EBP in fall prevention intervention, it is crucial that they have a goal to strive for. The older people’s goals and motivations involved the retention of basic skills for participating in daily life, being able to continue with leisure activities, and avoiding new falls. Most of the older people had found the motivation and the power to complete the intervention. The meaningfulness of the intervention, as well as several other psychological, physiological, and social benefits, were highlighted. They felt that by exercising, they were alive and well. Being able to participate in and enjoy daily life and physical activity contributed to positive emotions in the older people. One participant expressed his motivation for exercise and reminisced about the old days:

*first and foremost, I will be able to walk as a human being ... walking was my strength, walking a lot and dancing a lot. We were competition dancers, my wife and I … we were the first on the dance floor and the last ones to go off…* (Participant ID 3).

Another participant described how exercise had a positive impact on their appetite control and energy levels:

*I’m not sitting in this chair and nodding off so much, I have a little more energy and I think I have a little more appetite. For some reason, I have lost some appetite over the last few years, but it has come back a bit now. I can feel I’m hungry now, for a while I didn’t notice it. So, there was a time when I stopped exercising and sat nodding off, had no appetite...it’s gotten a little better now* (Participant ID 1).

The participants expressed both wishes and concerns for the future and the importance of exercise for their own benefit, but also for the benefit of their family members. One participant explained that she has to stay fit because she has a sick son with whom she walks:

*I have a son who is mentally ill … we tend to walk almost every day … his biggest problem is that he is isolating himself, and what I can contribute is to get him out for a walk ... what really motivates me is that I want to stay in the best possible shape since I have a son that I feel is dependent on me* (Participant ID 2).

Lack of motivation leads to low adherence and ultimately unsustainable treatments. Although most of the older people were motivated to participate in the intervention, some of them emphasized a lack of intrinsic motivation and enjoyment. Lack of motivation despite adequate knowledge was perceived as a barrier for engaging in fall prevention exercise. One of the participants put it this way:

*I manage everyday life, but it could be a lot better, but I am basically a little too lazy ... get up a little earlier in the morning, then go there to exercise and then the day has gone by ... I am aware about and understand what is positive or negative about it* (Participant ID 3).

#### Acquiring knowledge; knowledge as the basis for evidence-based practice

The participants considered the explanation and dissemination of research to be very important. Having knowledge about the evidence-based exercises facilitated participation in the treatment. The physiotherapists’ explanation of how to perform the exercises, together with information about health outcomes, made the intervention meaningful for the participant and enhanced their motivation. Furthermore, the participants appropriated the physiotherapists’ knowledge as a remedy for ambivalence. The physiotherapists` knowledge of the treatment, based on both evidence and clinical experiences, seemed to be crucial for the completion of the intervention. Understanding the purpose of the treatment made the rigorous fall-prevention exercises meaningful, according to some participants. The experience of a meaningful exercise also increased participants’ motivation. One participant described the importance of knowledge about the intervention and its benefits:

*It is very important that you know why you should do it and why you should achieve a good balance. It is particularly relevant once you’ve gotten older, because no one wants to fall* (Participant ID 6).

Although most of the older people experienced increased motivation and involvement in treatment after acquiring knowledge, this was not sufficient for all. Laziness and lack of joy over physical activity were highlighted as barriers that could not be overcome with knowledge alone. Some participants expressed a desire for fun activities to promote motivation for exercise. They preferred exercises that emphasize humor and pleasure instead of rigorously performed exercises.

*If it is possible, it should come up with something fun, such as balloon play at the end, after the exercises. Something different and easier than these rigorous exercises* (Participant ID 4).

For some, knowledge was perceived as just knowing, and was considered worthless in terms of motivation for the intervention. However, knowledge seemed to be transformed into wisdom to deal with challenges and ambivalence, as one participant stated:

*it wasn’t out of great desire or eagerness from me, but I am so sensible and there is so much health in it ... I have told the physiotherapist that it is not out of desire and interest that I am here, but I realize I need to do something* (Participant ID 4).

Through the acquisition of knowledge, older people become responsible for their own health. Successful empowerment provides increased adherence, as one of the participants explained:

*... if you haven’t trained in a month then the training is broken ... So that’s why I train at home, so I don’t get that shortness of breath ... it’s because of the bronchi and lungs* (Participant ID 10).

### The power of the therapist-participant relationship and the process of putting knowledge into action

The physiotherapist’s ability to establish and maintain a relationship with the older person during the exercise intervention seemed to be crucial for the physiotherapist to implement EBP and achieve a successful fall prevention intervention. The physiotherapist must build an equal partnership with the participants in which they form a team, establishing a common frame of reference as a starting point for cooperation. The participants focused on the ability to listen, have empathy, and be reflective. Thus, an important precondition for EBP is active listening to the older people’s narrative, including sensitivity to their cues and concerns. Accessible, attentive therapy, together with the physical and psychosocial environment, are important aspects in the establishment of an empowering atmosphere, understood as a recognition of the older people’s needs. The relationship is generally viewed as a force for good in its own right, but also as a means to be able to apply and fulfil the EBP.

#### The importance of mutual understanding

The participants described how the therapist could empower them through mutual understanding and inclusion and how this could go wrong if the therapist failed to empower the older people.

However, some participants did not feel that they had to be included in decision-making and trusted the therapist’s choices entirely.

Good collaboration, where the older people’s needs and interests are prioritized, was perceived as valuable by several participants. User participation contributes to acceptance and recognition and strengthens the relational bond between physiotherapists and their participants. When user participation was solicited, the therapist’s approach was taken seriously. The physiotherapist’s involvement and interest in the participants’ life contributed to compliance and a positive expectation that the treatment would help. For some participants, this approach led to an increased commitment to treatment. The participants expressed that the relationship was of great importance for their knowledge development and credence in the treatment. Most of the participants asserted that the power of the relationship between them and the physiotherapist is enhanced by trust and knowledge. One participant stated that the therapist’s manner and ability to communicate inspired trust in the participant:

*Trust is built by the way you present things. He managed in an ok way to sell his message to me in simple ways, it can be a glance and there can be ways to say things to justify why we should do it and how it affects the body* (Participant ID 3).

Another participant said:

*He was so safe and secure when he gave us assignments and it is contagious. He always said: this is not dangerous; you can do this ... it seemed very safe ... I trusted him and what he taught me* (Participant ID 14).

Individual support and praise during the performance gave participants the motivation and confidence to challenge their own boundaries. Progress gave the participants a feeling of mastery, which motivated them to continue. One participant expressed how the therapist motivated them by referring to their progress, comparing their current state with how they were before the intervention started:

*There was always progress, but sometimes I think it went slowly, then the physiotherapist asked me, ‘don’t you remember the first time we went out with crutches? You didn’t even dare to look straight ahead.’ So, then I realized that I had made progress* (Participant ID 7).

One participant expressed how knowledge, research, and clinical expertise must be integrated with their values. Everyone is different, and the therapist and the older person must therefore jointly arrive at the treatment that is best suited. Collaboration between the therapist and the participant made it possible to adapt the treatment to the individual’s wishes and goals. The older persons’ voice in the dialogue with the physiotherapist is a premise for goal achievement. The possibility for user participation motivated most participants to complete the intervention and achieve their goals for treatment. One participant described a process in which she partly participated in the decisions and chose exercises according to her preferences. She questioned her own motivation if she was not included in decision-making:

*I think it was a good thing that they took into account my opinion and asked about my goal for the training. I could answer that I was motivated to do this, and it has made me practice more, try more, to try and do what I have said … However, it depends on whether I would have been equally motivated if they did not ask* (Participant ID 8).

Dilemmas and tensions in practice might occur when therapists struggle to fulfil evidence-based tasks while at the same time maintaining the relationship with their participant and respecting their needs and preferences. Lack of understanding of the older persons’ wishes or challenges contributed to low satisfaction and low adherence to treatment. One participant experienced a loss of trust in the therapist and thus a break in the relationship, leading them to ignore the professional guidance:

*I think it was badly done that they didn’t listen to what I was saying. Because if I sit here on this bike and ride so hard for so long, I’m done … but, I didn’t care, I just did it at my own will* (Participant ID 13).

While the experience of the physiotherapist as an expert was met with resistance to the treatment, there were several participants who did not have the need or desire to participate in the treatment design. They expressed confidence in the therapist’s competence and responsibility to make informed decisions on behalf of the older person. Despite apparent power differences, one participant described how the therapist’s role as a driver had a positive impact on the intervention:

*Well, here I stand and have to do as you say ... there is nothing else to think about ... I did not deny one thing and they kept saying that I was good ... then I thought, is there someone who refuses and does not to do it*? (Participant ID 7).

#### Evidence balanced by understanding, empathy, and sympathy

The therapist’s ability to see and understand the individual was expressed as the foundation of a good physiotherapist and crucial for EBP. The therapist’s interpersonal expertise was highly valued by the participants. Therapists’ positive qualities, such as the ability to show empathy, sympathy, and understanding for the older people, strengthened their relationship with physiotherapists. The older people indicated that the physiotherapist’s clinical experience with the elderly population is crucial for understanding their challenges. One participant described his experience with inexperienced therapists in terms of how they communicate with and instruct older people:

*Inexperienced people are not as good at instructing, they progress too fast or talk too fast, or we do not understand what they say ... they may need to be trained in how the age affects us or what happens to us as we get older.... the experienced [therapists] have a different pace when they explain something, talk aloud and take it easier. They show the exercises properly. They know it must be a slow pace, especially in the beginning* (Participant ID 15).

Illness is experienced individually, and older people therefore have different needs. The older person’s specific characteristics, values, needs, and preferences are considered along with skills required in everyday life and its context. Individualization of treatment is about more than just adjusting the exercise level, and empathy was described as a tool to meet and tailor the treatment to the older person’s challenges. As one participant put it:

*Empathy is important too; without it something may go wrong. You have to get into things - me and the neighbor may want to experience things completely differently, and we would have different needs even if we were dealing with the same illness … and it is perhaps what a therapist with experience will find out more easily than* a *recently graduated one* (Participant ID 2).

In addition to the therapist’s sensitivity, the ability to understand and assess the older person’s needs through both verbal and non-verbal cues was highlighted as an important feature of the therapist. For the participants, this was crucial for a positive treatment experience, as well as for their sense of progress and development. One participant described how a skilled therapist should be able to “read” their needs, which in this case involved a sense of mastery:

*A good therapist is someone who controls something that you think you can’t, but senses it and doesn’t demand too much, and if you make a mistake or you get a little out of it then you see it is taken into account, and they don’t do anything about it* (Participant ID 10).

### Research is interwoven with successful therapy and is an integral component of it

For older people, clinical expertise and evidence-based knowledge are perceived as integrated with the therapist. The participants reported that, together with the older people’s preferences, the three components of the EBP model merge into each other, becoming a whole when the therapist translated knowledge into practice. Evidence-based knowledge was understood and utilized as a seal of approval, indicating a “guarantee of high quality” and assurance that the treatment would produce the desired effect on older people’s daily activities when adapted to their preferences, needs, and skills. Trust and credibility of communicated knowledge are important prerequisites for EBP. Knowledge and awareness of the outcomes were viewed as facilitators for the intervention.

#### In clinical practice, research makes sense

The participants described that they cannot distinguish evidence-based knowledge and other forms of knowledge without assistance from the therapist. The participants are largely positive about evidence-based knowledge and the effects that it has had for current treatments; even when participants are cynical about research or don’t feel that it reflects their experiences, research is associated with quality.

The participants acknowledged that the evidence-based knowledge became a part of the therapist’s daily practice and that all practice was based on research. Knowledge and evidence-based knowledge are perceived as inseparable if the therapist does not explicitly focus on the communication of research. One of the participants stated:

*… the research—it’s in the therapist’s head* (Participant ID 9).

Furthermore, the participant explained that they did not have the knowledge to determine if an intervention is evidence-based or not. However, the participant described a desire to understand the therapist’s clinical reasoning, and how this led to greater clarity about evidence-based knowledge:

*It puts this workout and what we do into a larger context that is not just about us and what I can achieve...it helps us to hear that this [is something] you will benefit from, because everything from experience and everything from research suggests it* (Participant ID 9).

However, one of the participants stated that, during an intervention, the focus of an older person is on participation, and that they do not care whether the intervention is evidence-based:

*Most of them are very old, 80 years or older and have some sort of injury. They exercise at a very low level. Nevertheless, they are so proud even though they almost do nothing right* (Participant ID 16).

Despite this statement, the participants were unambiguous about the relevance of research. For most of the participants, research was perceived as motivating, beneficial, and relevant to the individual’s challenges. Several participants drew parallels between the exercises and their daily activities, which emphasizes the importance of research dissemination and explanation of the exercises. As one of the participants stated:

*If someone had said do that and that and I didn’t realize the point, I wouldn’t have bothered to do it ... waving arms and legs with no intention is just nonsense* (Participant ID 2).

Furthermore, they described that knowledge about fall prevention is important for both the individual and the community. The participants acknowledged that research in medicine and health has developed and shaped today’s clinical practice and has been crucial for developments in fall prevention. One participant described how the approaches had changed from passive treatment to physical activity and exercise:

*Old people with femur fractures were put to bed ... That’s over now. Today they are in full training ... I must say that I am happy that I live in this time. Lying in bed would have been horrible* (Participant ID 7).

Although some of the participants indicated that research into fall prevention was not in line with their complex clinical symptoms, they were positive about research. However, one of the participants claimed that skepticism about research could be due to lack of knowledge and expertise. Nevertheless, most of the participants expressed credence in research, despite the inability to critically evaluate the source of knowledge.

#### Credibility of knowledge

The participants expressed a positive attitude towards research. The research outcomes can be seen as an innovation driver to improve quality of care. Research provides knowledge and increases understanding, which is an important resource for knowledge credibility. For the older people, research provides a sense of security. Several described it as a quality designation. Having “faith” is associated with uncertain knowledge and is not sufficient in clinical practice. Research is the only way to obtain knowledge. Explanations and evidence that the treatment produces results inspire credence and willingness to complete the treatment. One participant expressed greater confidence in evidence-based interventions and their potential to produce positive outcomes:

*It must be proven in research that it gives results ... if it is just theory then I am not so open to it, but if it is practice-based then I am quite open to accept the research* (Participant ID 12).

The participants considered research as a prerequisite for good professional practice. Nevertheless, clinical practice research must be integrated with the therapist’s experience and adjusted to the older people’s wishes, needs, and skills. The participants described how the various sources of knowledge in EBP complement each other, but also emphasized the importance of research. One participant cited the research in EBP as irreplaceable, stating that:

*Evidence-based knowledge must be included as an equilibrium or valuable addition to experience-based knowledge and user knowledge* (Participant ID 1).

The older people’s trust in research was attributed to the research process. The evidence demonstrating that the intervention is effective is based on thorough assessments, where “sloppiness” is omitted. One participant described how the researchers reached consensus and conclusions through professional discussions:

*Research is thoroughly evaluated. It’s not something you just write down and adopt. There are several [specialists] who consider and discuss it* (Participant ID 13).

## Discussion

To our knowledge, this is the first study that has explored the relations between evidence-based knowledge, clinical expertise, and patient values—from older people’s perspective in Norwegian primary health care. Overall, the findings demonstrate that EBP is predominantly perceived as positive quality assurance, signifying that practice is keeping up with changes in the field, and providing relevant practices for older people managing everyday life. EBP was referred to as the manifestation of a negotiation and learning process to create clinical knowledge in real-life contexts. The older people asserted that the experience of seamless, dynamic, and interactive transitions and the fusion of the three components of EBP increased their confidence and credence in the treatment. One of the main findings for the success of the fusion of EBP is the therapist’s relational competence.

There appears to be considerable consensus among researchers that EBP is based on a combination of three different knowledge sources: evidence-based knowledge, clinical expertise and the patient’s values [51]. The present study indicates that evidence-based knowledge is referred to as an integrated component in physiotherapy practice. Our participants disclosed that evidence-basedpractice is in the therapists’ mind, which includes the fusion between professional expertise and knowledge derived from research*.* Nilsen et al. [52] stated that, in practice, it is not obvious where knowledge is drawn from, as practice-based and evidence-based knowledge are intertwined—a statement which supports our findings. Furthermore, participants talked about the importance of empathy, citing the significance of therapists’ efforts to consider their opinions and ask them about their goals for training. These findings revealed how the fusion of EBP’s three elements is accomplished by the therapist in the act of approaching the older people, a process that was regarded positively by the participants.

The participants descriptions of the characteristics of EBP seem to correspond with three of the six elements in the WHO’s definition of quality: effective, patient-centered, and safe [53]. The EBP intervention was effective because most of the participants reported positive physical, psychological, and social changes following treatment. Furthermore, participants realized that the evidence-based fall prevention intervention they had completed was meaningful and effective for managing their daily life. The WHO defines effective care as the provision of evidence-based care services to those who need them [53]. In the present study, participants appreciated that the intervention enabled them to experience such positive changes as being able to walk or continue with leisure activities, in addition to preventing new falls*.* In order for EBP to be effective, therapists must facilitate older people’s adherence to treatment by honouring their preferences, which is of great importance for behavioral change [54, 55]. The World Health Organization [56] defines adherence as “*the extent to which a person’s behaviour corresponds with agreed recommendations from a health care provider*.” Adherence to therapy is a primary determinant of treatment success [56], and is related to an older people’s capacity and willingness to comply with a prescribed intervention [57]. However, it is important to distinguish, as Horne [58] highlights, between adherence and concordance in treatments. Adherence is the degree to which older people meets the prescription ordered by a health professional, and it can have a connotation of blame or guilt. Concordance, on the other hand, is the degree of agreement between the treatment prescribed by a health professional and the results achieved by the participant. For older people to achieve good adherence to a treatment, the treatment must fulfil three prerequisites: it must be acceptable, understandable, and personally manageable [59].

Despite good evidence regarding the positive effects of exercise programs for fall prevention [5, 6, 60], older people are less likely to meet these public health recommendations, and research has reported that low exercise adherence is common among older people after hospitalization [61]. In the context of physiotherapy, adherence involves attending all appointments, following medical advice, and undertaking the prescribed exercises at the frequency prescribed [62]. As per the findings of this and several other studies, the reasons for low exercise adherence among older adults are complex [63, 64]. Bernhardsson et al. [55] suggested that older people’s preferences are an important indicator of adherence, and their preferences should be integrated in evidence-based practice in physiotherapy. As shown in our findings, many participants wished to be involved in the management of their own challenges and told why they had to exercise, rather than simply when and how. This level of inclusion had a positive impact on participants’ motivation, as they felt that they should train more and often to keep their promises to themselves. Older people’s preferences can cover areas such as treatment methods, the level of involvement in clinical decision-making, and the type and amount of information they want from their clinician [65]. Preferences can be influenced by personal values and attitudes, health beliefs and expectations, and other cognitive, emotional, and relational factors [66]. Munro et al. [67] noted that the patient’s choice to accept an intervention is framed by the physiological and psychological effects of the intervention, as well as by the social and cultural structures in which the patient lives. One participant, a mother with a mentally ill son, cited her caring responsibilities as a motivator to exercise. Findings from the current study revealed that, to achieve high adherence, the EBP delivered by the therapist had to meet the older people’s need for confirmation, guidance, knowledge, and confidence that the exercises were effective and safe.

The degree to which health care quality can be defined as acceptable is strongly dependent on the service providers’ ability to meet the needs of the users, and to adapt to their expectations and perceptions [68]. In accordance with Worum et al. [25], EBP in fall prevention met the patients’ desire to cope with everyday life; in addition, they experienced more energy. Satisfactory EBP involves shared decision-making and patient-centered treatment. Shared decision-making can be an approach in which clinicians and patients make decisions together using the best available evidence [69]. Shared decision-making is the foundation of patient-centered care [70]. In shared decision-making, health care providers partner with patients to support patients in making health care choices consistent with their values and priorities. Shared decision-making involves mutual understanding between health care professionals and their patients. Furthermore, EBP must be appropriate and practical, which entails both acceptance of the therapist’s clinical expertise and evidence-based knowledge as well as consideration of the patient’s preferences. The participants in our study stated the importance of the health care provider’s communication. They communicated in a way that showed respect and sensitivity to the older people’s needs. In accordance with findings from a study conducted by Coulter [71], our participants stated that health care professionals had to share available treatment and care options along with their knowledge and experience, and participants reciprocate and share their knowledge, experience, and preferences [71]. To practice shared decision-making, therapists must make sure that patients understand the information they are provided, repeating information several times, as needed. One of our participants revealed the importance of therapists’ praise, support, and follow-up during the exercises for shared decision-making. The therapist’s praise inspires hope, which is important for effective treatment. However, person-centered care and shared decision-making does not mean that all decisions should be based on patients preferences alone; the patient does not always need to be the final arbiter of decisions [66, 72]. An unreflective emphasis on preferences can lead health care staff to think that person-centered care is unachievable in situations where they do not believe autonomous choice is appropriate. The participants in our study seemed to feel strongly that EBP should be person-centered. To be truly person-centered, it is important to both understand the older person’s perspectives and develop strategies in collaboration with them. Successful EBP satisfied the user’s demands and expectations for treatment. Similar to results from a study conducted by de Groot and Fagerström [73], our participants shared a strong motivation to engage in everyday activities and to live an autonomous life. The participants expressed a need for knowledge about ageing so that they would be able to prevent the challenges they face. Knowledge motivated them to maintain their functional status, and knowledge was thus considered as a preference. In line with results from a study conducted by Holli et al. [74], the participants in the current study stated that evidence and clinical-based knowledge must be provided to patients to encourage their hope that they could eventually cope with everyday life and functional and health challenges.

Delivering services that are safe is central to quality health care [75], and this corresponds well with our participants’ statements. Older people’s relationship with and trust in the therapist contributed to the perceived quality of health care. One participant stated that he felt safe and secure during the exercises, that he felt he could do the training, and that he trusted the therapist. The relationship between physiotherapist and the older person is very important for the outcome of treatment [76], and it is also a key factor in the practice of EBP. Relational competence is about communication, and it involves establishing and maintaining a relationship with the patient for the duration of treatment [77, 78]. Relational competency in patient- and person-centered care models focuses on the ability to listen, have empathy, be reflective, and know the self [32, 79]. Proper use of communication skills increases health care providers’ understanding of patients’ individual needs, values, and perspectives, provides patients with the information they need to participate in their care, and builds trust and understanding between health care providers and patients [74, 80]. Furthermore, there is a need for practical guidance on how to incorporate this advice into older people’s daily lives. As expressed by one of the participants, one way therapists can ensure adequate communication and guidance is to not talk too fast or demand too much. Another is to cultivate adequate knowledge of what happens when one gets older*.* Thus, health care providers need to be effectively trained in communication skills in a systematic way, for example, through practice dialogues [80].

The participants in the current study considered the service they were offered as beneficial, supporting the combination of individual, clinical, and professional expertise with the best available external evidence to produce practices that lead to positive outcomes for patients [81]. This is in accordance with the definition of EBP [3]. As shown in the present study, the experience of autonomy and attitudes and beliefs about the cost and benefits of exercise in later life can influence an older person’s activity level. The participants reported that they believed in the benefit of exercise and that acquiring knowledge was of great importance for their motivation as it assured them of the intervention’s proven efficacy. This is in contrast to findings from another study [81] that showed that many older people have misconceptions about the ageing process and think that physical exercises are not beneficial for them [82]. However, previous studies have suggested several factors that can help to increase exercise adherence among older people, such as information about the benefits of physical activities and a focus on increasing self-efficacy [82, 83]. These findings are consistent with those of the present study. Various motivational and relational determinants were found to influence adherence practices. For instance, Sandlund et al. [84] noted that it is preferable to consider the experiences and desires of the older individual when planning and prescribing fall preventive exercises. Worum et al. [25] emphasized the importance of tailored intervention for older people in the Otago fall-prevention exercise program. These statements are in line with our findings. The therapist’s qualities and abilities to understand and be sensitive to each individual older person’s physical and mental needs was described as crucial for adherence. Sandlund et al. [84] explained that a therapist’s characteristics may also play a role in adherence to an individually-tailored exercise program. Findings from their study demonstrate that confirmation was secured through visible results of practical activities, by assessment and evaluation of performance, or simply by feelings of achievement, all of which are in line with our findings [84].

The therapist-participant relationship might entail differences in power. The older person needs the knowledge of the therapist because knowledge controls what the older person can or cannot do*.* According to Maynard-Moody and Musheno [85], welfare workers are powerful because they act as both state agents, vested with formal power, and as citizens’ agents, vested with the power to decide what is best for the citizen, which can be seen as a soft type of paternalism. Fillit et al. [86] argue that paternalistic decision-making rarely or never can be justified in non-emergency situations with individual patients; therefore, an autonomous, patient-centered care model is preferable for both patients and therapists when performing EBP.

This study has certain limitations that must be considered when interpreting the results. Limitations stem from the characteristics of the sample and the nature of qualitative methods. The material from an interview is not a one-and-only truth; instead, it is highly dependent on chosen perspectives. Furthermore, although there are several interventions and multifactorial interventions to prevent falls in older people [5], this article only addresses older people who have participated in exercise-based fall prevention intervention. It should therefore be noted that this paper addresses only a limited percentage of older persons at risk of falls. The study was carried out on a relatively small group of 16 participants from different regions in Oslo, Norway. However, in terms of trustworthiness and transferability, the description of the context and the participants provides the readers with the opportunity to assess whether the findings are transferable to similar contexts. Ten to fifteen interview transcripts are an ideal number for qualitative analysis [87]. Only two of the participants were not ethnic Norwegian and all participants had middle or high education level. The respondents were chosen on the basis of the position they hold as important stakeholders in fall-prevention exercises. This suggests that similar understandings would likely be found in other fall-prevention programs or settings—at least those consisting of older people who had fallen and then participated in different Norwegian fall-prevention programs—but probably with slight differences depending on emphasis.

The findings impact on clinical practice and associated training for practitioners that the clinicians must ingest the role as a mediator for the negotiations of different sources of knowledge as evidence-based knowledge and clinical expertise in relation to the older people’s preferences. These findings may help us to understand the importance of clinician’s relational competence in order to succeed with EBP. The clinician’s ability to integrate and negotiate the different sources of knowledge will reduce the tension between the various elements of EBP (evidence-based knowledge, clinical expertise, patient values) and therefore be crucial for successful fall prevention intervention. Related research should also address the importance of the therapist’s role as a motivator regarding adherence and behavior change regarding fall prevention intervention.

## Conclusions

Older peoples’ perspectives on the relations between the EBP model’s three dimensions—evidence-based knowledge, clinical expertise, and patient values—disclosed a broad range of understandings of EBP in fall prevention. Evidence-based knowledge was understood and utilized as a seal of approval with a “guarantee of high quality,” and it was found that fall-prevention intervention will give the older people the desired effect on their daily activities if it is adapted to their preferences, needs, and skills. Older people’s preferences, knowledge, motivation, abilities, and skills influence them to varying degrees to make important health-related decisions and are therefore crucial for their participation in therapy. The participants considered clinical expertise and evidence-based knowledge to be integrated with the therapist. Relational competence involves a physiotherapist’s skills in communication and their ability to establish and maintain a satisfactory relationship with the participant during EBP. Making sense in evidence-based fall prevention represents the negotiation between different types of knowledge and contexts, constituting a discursive practice. Future studies could fruitfully explore this issue further in different contexts such as hospitals and institutes.

## Supplementary information

**Additional file 1.** Interview guide. Interview guide used during the interviews.

**Additional file 2.** COREQ. Consolidated criteria for reporting qualitative research: a 32-item checklist for interviews and focus groups

**Additional file 3.** GRIPP2-SF. Guidance for Reporting Involvement of Patients and the Public

## Data Availability

The dataset supporting the conclusions of this article can be obtained by contacting the first author, Hilde Worum.

## Abbreviations

EBP: Evidence-based practice
KT: Knowledge Translation
SPPB: Short Physical Performance Battery
