# Supplementary material for: Reflections of older people about their experience of fall prevention exercise in the community- a qualitative study exploring evidence-based practice
Source: BMC Public Health. 2020 Nov 9;20:1671. doi: 10.1186/s12889-020-09630-4 (PMC7650178; doi:10.1186/s12889-020-09630-4)
Supplement: Supplementary file 3 — Additional file 3. GRIPP2-SF. Guidance for Reporting Involvement of Patients and the Public [file 12889_2020_9630_MOESM3_ESM.docx]

Table 2

GRIPP2 short form

| **Section and topic** | **Item** | **Reported on page No** |
| --- | --- | --- |
| 1: Aim | Report the aim of PPI in the study  The aim of this study was to explore older patients’ reflections and experiences about EBP in fall prevention, so as to close the know-do gap in fall prevention. We aimed to explore, from a patient’s perspective, the relations between three dimensions of the EBP model: evidence-based knowledge, clinical expertise, and patient values. | 5 |
| 2: Methods | Provide a clear description of the methods used for PPI in the study  We conducted semi-structured interviews with 16 community-dwelling older patients.Their respective physiotherapists conducted initial contact with the informants, and was then recruited by the first author. | 6-7 |
| 3: Study results | Outcomes—Report the results of PPI in the study, including both positive and negative outcomes  PPI contributed to the study in several ways, including:  -collating evidence about patient opinions about, the relations between three dimensions of the EBP model: evidence-based knowledge, clinical expertise, and patient values.  - the patients highlighted the importance of their preferences, knowledge, motivation, abilities, and skills influence them to varying degrees to make important health-related decisions, and are therefore crucial for the patients’ participation in the intervention.  -Understanding the views and expectations of older people is fundamental as well as facilitating person-centred care, because respect for individual diversity is a basic pillar in the approach of EBP.  -Understanding the views and expectations of older people is fundamental and provides health and social care professionals with a common understanding of the principles of appropriate individual health care.  -Findings of this study might contribute to improved strategies that can inform policy makers, educators, clinicians, future researchers, and older adults. | 5 |
| 4: Discussion and conclusions | Outcomes—Comment on the extent to which PPI influenced the study overall. Describe positive and negative effects  The physiotherapists who were responsible for initial contact with the patients were informed about the study, background, the aim, as well as presentation of the interview guide, which they had opportunity to provide input to.  The perspective of **older** patients is fundamental in EBP, as they have their own individual and meaningful rationale for exercising. Considering the **views** of older patients is important for identifying their needs, which in turn helps providers to develop appropriate and responsive services.  The research process in qualitative method is emerging / evolving. This means that the planned research process and associated research questions, collection method, interview guide are only tentative and possible to change as the research is ongoing. In combination with PPI, it gave us the opportunity to elaborate follow-up questions in the informants' responses relevant to research topics throughout the study. The participants in this study provided rich responses to the research phenomenon, which provided an adequate exploration of the research phenomenon. | 6-7  4  None |
| 5: Reflections/critical perspective | Comment critically on the study, reflecting on the things that went well and those that did not, so others can learn from this experience  Rapid recruitment of available and relevant informants was an important factor in terms of that it is a PhD project and thus time-limited. In this project, recruitment took place without problems, but as in all research, recruitment can be a challenge.  **The following text is taken directly from the article:**  *To maintain rigour throughout the analysis, features of trustworthiness were established according to Lincoln and Guba’s criteria of soundness. The criterion of credibility was met through open-ended questioning and prolonged engagement with the data, and by providing a detailed description of the methods. The criterion of transferability was fulfilled by presenting detailed and in-depth descriptive data from the participants’ quotes. To meet the criterion of dependability, each transcription was independently read, checked, and coded by the first and the last authors; final interpretations were reached via agreement among all five authors. The criterion of confirmability was fulfilled by providing rich quotes from the participants depicting each emerging theme. Furthermore, the consolidated criteria for reporting qualitative studies (COREQ) and Guidance for Reporting Involvement of Patients and the Public (GRIPP2-SF) were considered for reporting the current study.*  *This study has certain limitations that must be considered when interpreting the results. Limitations stem from the characteristics of the sample and the nature of qualitative methods. The material from an interview is not a one-and-only truth; instead, it is highly dependent on chosen perspectives. The study was carried out on a relatively small group of 16 patients from different regions in Oslo, Norway. However, in terms of trustworthiness and transferability, the description of the context and the participants provides the readers with the opportunity to assess whether the findings are transferable to similar contexts. Ten to fifteen interview transcripts are an ideal number for qualitative analysis. Only a few of the patients (n = 2) were not ethnic Norwegian. The respondents were chosen on the basis of the position they hold as important stakeholders in fall-prevention exercises. This suggests that similar understandings would likely be found in other fall-prevention programs or settings—at least those consisting of older people who had fallen and then participated in different Norwegian fall-prevention programs—but probably with slight differences depending on emphasis.* | None  11-12  31-32 |

PPI=patient and public involvement
